# Carbon Wrapped Ni_3_S_2_ Nanocrystals Anchored on Graphene Sheets as Anode Materials for Lithium-Ion Battery and the Study on Their Capacity Evolution

**DOI:** 10.3390/nano8100760

**Published:** 2018-09-26

**Authors:** Xianggang Guan, Xuehua Liu, Binghui Xu, Xiaowei Liu, Zhen Kong, Meiyun Song, Aiping Fu, Yanhui Li, Peizhi Guo, Hongliang Li

**Affiliations:** 1 Institute of Materials for Energy and Environment, Qingdao University, Qingdao 266071, China; wl_gxg@163.com (X.G.); liuxuehua@qdu.edu.cn (X.L.); xubinghuiqdu@qdu.edu.cn (B.X.); xiaoweiliu1120@163.com (X.L.); KZ577718484@163.com (Z.K.); pzguo@qdu.edu.cn (P.G.); 2 College of Materials Science and Engineering, Qingdao University, Qingdao 266071, China; m13021668805@163.com (M.S.); apfu@qdu.edu.cn (A.F.); 3 College of Electromechanic Engineering, Qingdao University, Qingdao 266071, China; liyanhui@qdu.edu.cn

**Keywords:** nickel sulfide, carbon coating, graphene, wrapping effect, ex-situ TEM

## Abstract

Ni_3_S_2_ nanocrystals wrapped by thin carbon layer and anchored on the sheets of reduced graphene oxide (Ni_3_S_2_@C/RGO) have been synthesized by a spray-coagulation assisted hydrothermal method and combined with a calcination process. Cellulose, dissolved in Thiourea/NaOH aqueous solution is chosen as carbon sources and mixed with graphene oxide via a spray-coagulation method using graphene suspension as coagulation bath. The resulted cellulose/graphene suspension is utilized as solvent for dissolving of Ni(NO_3_)_2_ and then used as raw materials for hydrothermal preparation of the Ni_3_S_2_@C/RGO composites. The structure of the composites has been investigated and their electrochemical properties are evaluated as anode material for lithium-ion batteries. The Ni_3_S_2_@C/RGO sample exhibits increasing reversible capacities upon cycles and shows a superior rate performance as well. Such kinds of promising performance have been ascribed to the wrapping effect of carbon layer which confines the dislocation of the polycrystals formed upon cycles and the enhanced conductivity as the integration of RGO conductive substrate. Discharge capacities up to 850 and 630 mAh·g^−1^ at current densities of 200 and 5000 mA·g^−1^, respectively, are obtained. The evolution of electrochemical performance of the composites with structure variation of the encapsulated Ni_3_S_2_ nanocrystals has been revealed by ex-situ TEM and XRD measurements.

## 1. Introduction

Lithium-ion batteries (LIBs) are considered as the most important electrochemical energy storage devices to power electric vehicles, hybrid vehicles and consumer electronics [1,2,3,4]. However, the graphite anode that has been used for LIBs cannot meet the increasing requirements for high-energy density LIBs because of its low theoretical capacity (372 mAh·g^−1^) [5]. Therefore, numerous efforts have been made to exploit alternative anode materials with high-energy density and long cycle life. Transition metal dichalcogenides [6]. (e.g., nickel sulfides) possess a large number of active sites for Li^+^ insertion [7]. This kind of materials holds a great promise for developing high-energy density anode materials for LIBs [8]. Thus, several nickel sulfides with different stoichiometric ratios of nickel and sulfur, for example, NiS, Ni_3_S_2_, NiS_2_, Ni_3_S_4_, Ni_7_S_6_, Ni_9_S_8_, have been reported for energy storage and conversion applications, ranging from supercapacitors [9,10,11,12], electrocatalysis [13] to LIBs [14,15,16,17,18]. Among them, Ni_3_S_2_ is featured with rich abundance and low cost. However, its poor electric conductivity and aptness to structure pulverization upon insertion/extraction of Li^+^ lead to unsatisfactory cyclic stability and rate performance, hindering its practical applications. To circumvent these shortcomings, two kinds of strategies normally are explored. (1) Diminishing the size of Ni_3_S_2_ to nano ranges and combining with a carbon coating to alleviate its structure pulverization; and (2) anchored onto or integrated into a flexible substrate of high conductivity to form a three-dimensional structure with buffer void. Therefore, nanoparticles of Ni_3_S_2_ have attracted intense concerns because the zero-dimensional (0 D) structure can provide facile channels for Li^+^ insertion and extraction and also easy to be wrapped by a carbon layer to prevent the volume changes. As a kind of novel two-dimensional carbon structure, graphene has been found to be an ideal two-dimensional (2D) substrate for improving the electrical conductivity of the composites. Consequently, composites of Ni_3_S_2_ nanocrystals coated with a thin carbon layer and anchored onto a graphene sheet will be desirable for improving their electrical conductivity and structure stability against cycling. Many endeavors have been devoted to such a structure. Meanwhile, appropriate carbon sources and facile methods for achieving such kind of composite materials, especially in which the graphene sheets assemble into a three-dimensional structure, are still scanty [19]. Biomass such as cellulose and chitosan are found to be a desirable source for forming amorphous carbon upon carbonization, which can be used as carbon coating and/or the binder for nanoparticles and graphene sheets. Surfactants normally are useful for tuning the size and shapes in materials synthesis, meanwhile, they can also be used as ultrathin coating layer due to their amphiphilic properties and then act as an uncommon precursor for facilitating the uniform carbon coating of the substrates upon calcinations [20].

In this work, nanosized Ni_3_S_2_ crystals coated with an ultrathin carbon layer and anchored on graphene sheets with 3-dimensional structure (named as Ni_3_S_2_@C/RGO) have been achieved by using a spray-coagulation assisted hydrothermal method. The spray-coagulation strategy was used to prepare nano-sized cellulose sheets by mimicking the fabrication process of viscose fiber in spinning industry [21,22]. The cellulose worked as both binder and carbon source for the assembly of 3D graphene framework. The carbon/RGO framework has been utilized as substrates for the Ni_3_S_2_ nanocrystals and played an important role in accommodating the volume change of Ni_3_S_2_ crystals during charging/discharging. Owing to the wrapping effect of carbon layer, the flexibility and the enhanced conductivity of the graphene substrates, the capacity decay due to pulverization of the Ni_3_S_2_ nanocrystals upon Li^+^ insertion/extraction was relieved. As a consequence, the Ni_3_S_2_@C/RGO composite delivered an excellent electrochemical performance in terms of cycling stability and rate capacity with an extremely high capacity of 950 mAh·g^−1^ at the current density of 100 mA·g^−1^ which is double of the theoretical capacity for Ni_3_S_2_. Interestingly, a capacity increasing phenomenon was observed during the cycling that was found to be associated with structure evolution of the Ni_3_S_2_ nanocrystals upon cycles.

## 2. Experimental Section

### 2.1. Materials

Anhydrous ethanol, acetic acid (CH_3_COOH, 36.0–38.0 wt %), nickel nitrate hexahydrate (Ni(NO_3_)_2_·6H_2_O), sodium sulphide (Na_2_S·9H_2_O), thiourea (TU), cetyltrimethyl ammonium bromide (CTAB) and sodium hydroxide (NaOH) are of AR grade and were purchased from Sinopharm and used without further purification. Cellulose with a viscosity-average molecular weight of 8.5 × 10^4^ was obtained from Hailong Chemical Fiber Co. (Weifang, China). Graphene oxide (GO) was kindly provided by the Sixth Element Ltd. (Changzhou, China). Deionized water was used throughout this work.

### 2.2. Sample Preparation

#### 2.2.1. Synthesis of Cellulose Sol

**Scheme 1:**
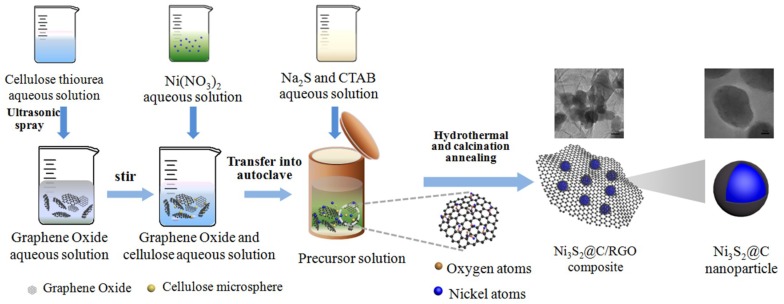
Schematic illustration showing the synthesis of Ni_3_S_2_@C/RGO composites.

The preparation procedure of samples is schematically illustrated in Scheme 1. 0.9 g of TU and 1.8 g of NaOH were dissolved in 21 mL of water to obtain a TU/NaOH solution. Then, 0.05 g of cellulose was added to the solution under stirring for 1 h. The suspension was subsequently transferred into a refrigerator and kept at 263 K for 10 h [23]. A homogenous cellulose sol in TU/NaOH aqueous solution was obtained after melting at room temperature and was named as sol A).

#### 2.2.2. Synthesis of Ni_3_S_2_@C/RGO Composite

0.05 g of GO was dispersed in 54 mL of 5 wt % acetic acid aqueous solution followed by irradiation with a high power sonicator for 2 h. Sol A was dispersed into the GO solution via an ultrasonic spraying process and then kept stirring for 2 more hours, obtaining a suspension labeled as B. After that, two batches of solution, that is, 1.5 mmol of Ni(NO_3_)_2_·6H_2_O dissolved in 22.5 mL of water and 6 mmol of Na_2_S·9H_2_O and 0.05 g of CTAB dissolved together in 22.5 mL of water, were poured into B suspension in sequence under stirring, resulting in a mixture of precursors. After the addition of 10 mL of ethanol, the precursor mixture was transferred into a 150 mL Teflon-lined autoclave and heated at 200 °C for 16 h. The hydrothermal product was washed with ethanol and water thrice and then was freeze-dried for two days. The resulted solid was calcinated at 700 °C for 2 h with a heating rate of 5 °C min^−1^ in a tube furnace under an atmosphere of H_2_/Ar mixture (1:9, *v*/*v*). After cooling to room temperature, a black solid product was obtained and is designated as Ni_3_S_2_@C/RGO. For comparison purposes, the following three control samples were prepared. Pristine Ni_3_S_2_ was prepared using the same procedure as Ni_3_S_2_@C/RGO except for replacing cellulose/GO suspension with water. Samples Ni_3_S_2_@C was prepared as Ni_3_S_2_@C/RGO without GO, while sample Ni_3_S_2_@RGO was obtained without cellulose. The synthesis mechanism of the Ni_3_S_2_@C/RGO composites has been illustrated in Scheme 1.

### 2.3. Characterization

X-ray diffraction (XRD) patterns were recorded on a Ultima IV X-ray diffractometer with Cu-Kα radiation (*λ* = 0.15418 nm) (Rigaku Corporation, Tokyo, Japan). Raman spectra were collected using an inVia Plus Micro-Raman spectroscopy system equipped with a 50 mW DPSS laser at 532 nm (Renishaw plc., Gloucestershire, UK). N_2_ isotherms were measured on a Autosorb-IQ-MP/XR surface area and pore analyzer (Quantachrome Instruments, Boynton Beach, FL, USA). The specific surface areas were estimated with the Brunauer-Emmett-Teller (BET) method from the N_2_ adsorption data in the relative pressure range of P/P_0_ = 0.05–0.35. The pore size distributions were calculated using the Barrett-Joyner-Halenda (BJH) model applied to the desorption branches of the isotherms. Thermo gravimetric analysis (TGA) was performed on a TGA-2 thermal gravimetric analyzer (Mettler Toledo International Inc., Greifensee, Switzerland) in air with a heating rate of 10 °C min^−1^. The morphologies and the microstructures of the samples were examined by using a JSM-7800F scanning electron microscope (SEM) (JEOL Ltd., Tokyo, Japan) equipped with an energy dispersive X-ray (EDX) (Oxford Instruments, Oxford, UK) and a JEM-2100Plus transmission electron microscope (TEM) (JEOL Ltd., Japan).

### 2.4. Electrochemical Measurements

A mixture of 80 wt % active materials, 10 wt % carbon black and 10 wt % polyvinylidene fluorides (PVDF) were dispersed into N-menthyl-2-pyrrolidinone (NMP). Afterwards, the obtained slurry was cast onto a copper foil and dried in vacuum at 110 °C for 10 h to remove excess solvent and pressed under pressure of 10 MPa. The active materials loaded on the electrode were approximately 0.6–1.2 mg·cm^−1^. CR2016 type coin cells were assembled in a highly pure Argon-filled glove box using pure lithium as anode, 1 mol L^−1^ LiPF_6_ (Capchem Technology Co., Ltd., Shenzhen, China) as electrolyte and Celgard 2400 membrane as separator. The cells cycled at the potential range of 0.01–3 V on a Land testing system and calculate the specific capacity by the weight of the composites electrode materials Ni_3_S_2_@C/RGO. Cyclic voltammetry (CV) measurements were performed under a scan rate of 0.2 mV·s^−1^ between 0.01 and 3 V. Electrochemical impedance spectra (EIS) was measured at a frequency range from 100 KHz to 1 Hz with a signal amplitude of 5 mV.

## 3. Results and Discussions

### 3.1. XRD

**Figure 1:**
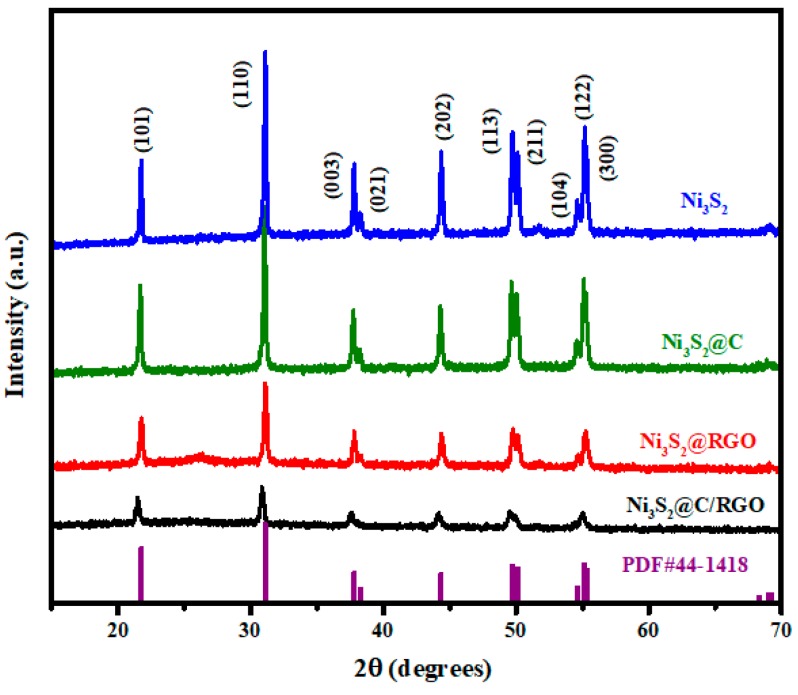
XRD patterns of Ni_3_S_2_, Ni_3_S_2_@C, Ni_3_S_2_@RGO and Ni_3_S_2_@C/RGO thermally treated at 700 °C.

Figure 1 shows the XRD patterns of pristine Ni_3_S_2_ and that of the composite materials. All samples show quit similar patterns with peaks at two theta of 21.7, 31.1, 37.8, 44.3, 49 and 55.1 degrees. They can be assigned to the (101), (110), (003), (202), (113) and (122) facets of crystalline Ni_3_S_2_ (PDF # 44-1418). The XRD pattern of sample Ni_3_S_2_@C matched well with that of pristine Ni_3_S_2_. While, a weak and broad peak at two theta of about 25.8 degrees is observed for sample Ni_3_S_2_@RGO, corresponding to the characteristic (002) planet of RGO. Such a speculation has been confirmed by comparing it with the XRD patterns of GO and RGO as shown in Figure S1a [24,25,26]. For sample Ni_3_S_2_@C/RGO, a weak peak is observed at two theta of 51.7 degrees and it can be indexed to metallic nickel (PDF # 65-0380) formed during the thermal treatment process in a H_2_/Ar atmosphere. Such a result has been confirmed further by the XRD patterns of the Ni_3_S_2_ samples thermally treated at 700 and 750 °C (Figure S1b). The sample thermally treated at 750 °C shows more obvious metallic nickel related peaks than other samples. In this work, we therefore selected the sample thermally treated at 700 °C for further investigation.

### 3.2. SEM and TEM

**Figure 2:**
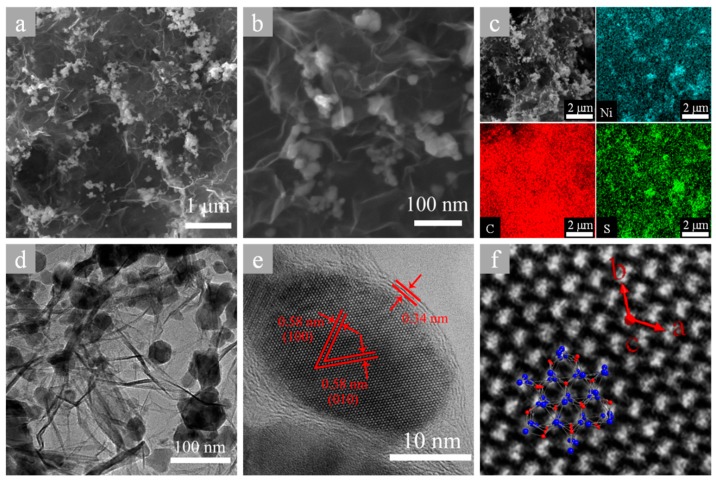
SEM images (**a**,**b**); EDS mapping (**c**); TEM image (**d**); and HRTEM images (**e**,**f**) of Ni_3_S_2_@C/RGO.

Pictures a and b of Figure 2 show the SEM images of Ni_3_S_2_@C/RGO composites. Ni_3_S_2_ particles anchored on RGO sheets can be clearly observed. The EDX elemental mappings as shown in Figure 2c clearly confirm the existence of Ni, S and C. Figure 2d shows the TEM image of Ni_3_S_2_@C/RGO. It can be seen that nanoparticles are distributed on RGO sheets uniformly, which is in agreement with the SEM results. Figure 2e shows the HRTEM image of a single Ni_3_S_2_ nanocrystal. A crystalline lattice of 0.58 nm can be calculated based on the measured fringes, which can be indexed to the (100) planes of hexagonal Ni_3_S_2_ phase. The simulation image of Ni_3_S_2_ nanocrystals (CIF # 65-4935) has been depicted in Figure 2f to help determine the base coordinates. Interestingly, it can be clearly seen that the Ni_3_S_2_ nanocrystal is wrapped by a thin layer with thickness of few nanometers. The wrapping layer show obviously different lattice fringes with that of the Ni_3_S_2_ nanocrystal. An inter-planar spacing of 0.34 nm has been calculated and can be assigned to the (002) lattice planes of graphite. Such a structure indicated that the Ni_3_S_2_ nanocrystal is wrapped with a graphite thin layer and such a carbon layer may help avoid crumbling of the Ni_3_S_2_ nanocrystal and influence the electrochemical performance of the composite. Such a speculation has been verified by the following electrochemical property studies. The formation process of the carbon layer has been described in detail in the supporting information section (see SI and Scheme S1 and Figure S2 therein).

SEM images of control samples of Ni_3_S_2_, Ni_3_S_2_@C and Ni_3_S_2_@RGO are presented in Figure S3 for comparison. It can be seen that the particle sizes decreased in sequence of Ni_3_S_2_, Ni_3_S_2_@C and Ni_3_S_2_@RGO. Such a result demonstrated that the presence of carbon materials influences the growth of the Ni_3_S_2_ particles. Quite similar morphology with that of Ni_3_S_2_@C/RGO has been observed for Ni_3_S_2_@RGO control sample, in which the Ni_3_S_2_ nanocrystals were well dispersed on RGO sheets. Figure S4 shows the TGA curve of Ni_3_S_2_@C/RGO composite conducted in air up to 700 °C, during which the C/RGO moieties was oxidized to form gaseous species and the Ni_3_S_2_ was oxidized to NiO. The weight increase before 500 °C can be due to the oxygen absorption reaction of Ni_3_S_2_, while the significant weight loss event between 500 and 600 °C is due to the burning off of C/RGO and decomposition of sulfide. After a calculate based on the TGA data, it can be demonstrated that the composite contained about 87 wt % of residual and 13 wt % of C/RGO.

### 3.3. Raman Spectra

**Figure 3:**
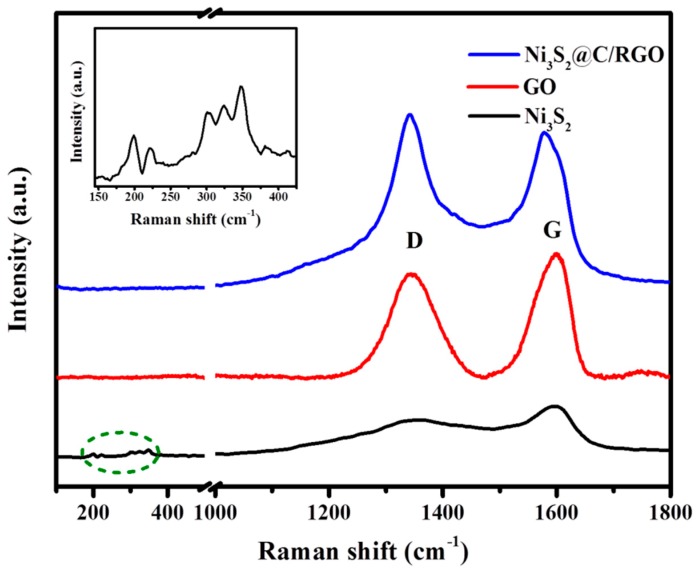
Raman spectra of Ni_3_S_2_@C/RGO, GO, Ni_3_S_2_ and the characteristic peaks of Ni_3_S_2_ (the inset).

The Raman spectra of Ni_3_S_2_, GO and Ni_3_S_2_@C/RGO samples in the frequency range of 100–1800 cm^−1^ are shown in Figure 3 and Figure S5. The peaks at 182, 199, 221, 301, 324 and 347 cm^−1^ coincide with the characteristic Raman peaks of Ni_3_S_2_ as reported in literatures [27,28]. The peak at about 1585 cm^−1^ (G band), assigned to an E_2g_ mode of graphite, it is related to the vibration of the sp^2−^ bonded carbon atoms in a two-dimensional hexagonal lattice, while the peak at about 1325 cm^−1^ (D band) is easily influenced by the lattice distortion (defects) in the hexagonal graphitic layers or atomic substitution (doping) [29,30]. The control sample of pristine Ni_3_S_2_ nanocrystals exhibited also those two bands, the existence of carbon materials. The carbon related Raman signal can be ascribed to the covering layer of carbon onto the Ni_3_S_2_ nanocrystals as has been revealed by TEM images of Figure 2 and Figure S2.

Obviously, The *I_D_*/*I_G_* intensity ratio in the Ni_3_S_2_@C/RGO (1.11) increased in comparison to those of Ni_3_S_2_ (0.78), GO (0.84) and Ni_3_S_2_@RGO (1.03). The high *I_D_*/*I_G_* ratio of Ni_3_S_2_@RGO can be attributed to the increasing of disordering of the RGO in the composite, due to the intercalation of Ni_3_S_2_ between the RGO sheets, while that for Ni_3_S_2_@C/RGO can be ascribed to both the disordering of the RGO sheet and the presence of amorphous carbon derived from the carbonization of cellulose [31,32,33].

### 3.4. Electrochemical Performance

**Figure 4:**
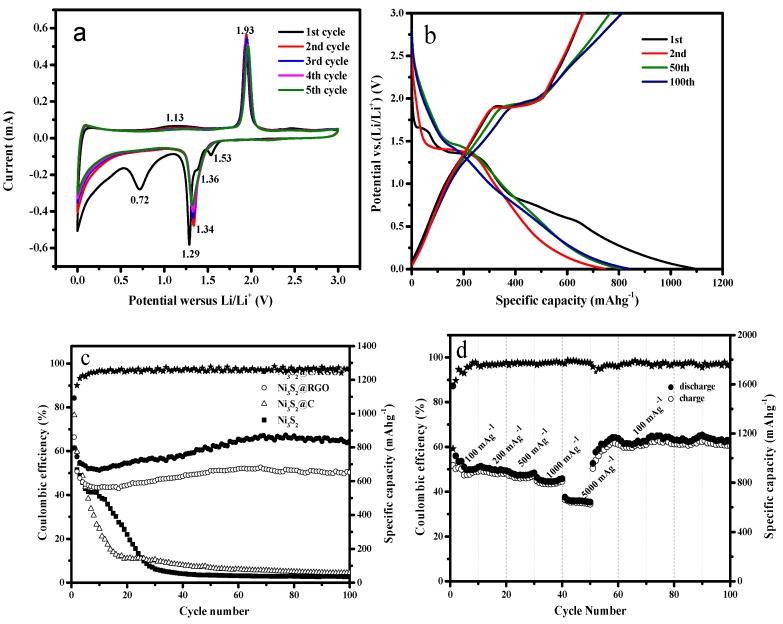
The electrochemical performance of the Ni_3_S_2_@C/RGO electrodes. (**a**) Cyclic voltammograms of the Ni_3_S_2_@C/RGO composite at a scan rate of 0.2 mV·s^−1^; (**b**) Charge-discharge voltage profiles of the Ni_3_S_2_@C/RGO materials at a current density of 200 mA·g^−1^; (**c**) Charge-discharge cycling of these electrodes; (**d**) Rate performance of the Ni_3_S_2_@C/RGO electrode at different current densities from100 to 5000 mA·g^−1^.

Electrochemical measurements were conducted to investigate the performance of these samples as anode materials of LIBs based on coin-type cell with Li foil as counter electrode. Figure 4a shows the cyclic voltammetry (CV) curves of Ni_3_S_2_@C/RGO electrode recorded in a potential range of 0.01–3.0 V (vs. Li^+^/Li) at a scan rate of 0.2 mV·s^−1^. The reduction peaks at 0.72, 1.29 and 1.54 V in the first cathodic sweep are attributed to the insertion of Li^+^ (4Li^+^ + 4e^−^ + Ni_3_S_2_ = 3Ni + 2Li_2_S, corresponding to a theoretical capacity of 445 mAh·g^−1^), as well as the formation of a solid electrolyte interface (SEI) layer on the active material surface. These cathodic shoulder peaks disappear and the main peak shifted to 1.34 V in the second scanning cycle owing to the activation of the material [32,34]. The oxidation peak at 1.13 V in the first anodic scan, which is the counterpart of the reduction peak at 0.72 V in the first cathodic sweep, disappeared after the first cycle. Meanwhile, the oxidation peak at 1.93 V due to the extraction of Li^+^ (3Ni + 2Li_2_S = Ni_3_S_2_ + 4Li^+^ + 4e^−^) increases upon scans [35]. It should be noted that the CV curve of the second round consist well with that of the fifth cycle, suggesting an excellent reversibility of the Ni_3_S_2_@C/RGO electrode for Li^+^ storage. Figure 4b shows the charge-discharge curves of Ni_3_S_2_@C/RGO electrode at a current density of 200 mA·g^−1^ under a voltage range of 0.01–3.0 V. In the first discharge process, three voltage plateaus at 0.60, 1.35 and 1.65 V, corresponding to the first reduction peaks in the CV scan, can be observed. During the subsequent cycles, the charge and discharge platforms are observed at 1.38 and 1.91 V, respectively. After 100 cycles, the electrode delivered a reversible capacity of 820 mAh·g^−1^ with a weak voltage platform. A comparison of the cycling performance for Ni_3_S_2_, Ni_3_S_2_@C, Ni_3_S_2_@RGO and Ni_3_S_2_@C/RGO electrodes at current density of 200 mA·g^−1^ is depicted in Figure 4c. The Ni_3_S_2_ and Ni_3_S_2_@C samples clearly experienced a dramatic decay in capacity with cycling and their capacity decreased to few tens mAh·g^−1^ after 30 cycles. Samples Ni_3_S_2_@RGO and Ni_3_S_2_@C/RGO, however, displayed a high reversible capacity, especially for sample Ni_3_S_2_@C/RGO, which exhibited a maximum discharge capacity of 1091 mAh·g^−1^ (0.67 mAh·cm^−2^) in the first cycle and a charge capacity of 750 mAh·g^−1^. The capacity loss can be ascribed to the formation of SEI film [36]. Besides, the Ni_3_S_2_@C/RGO composite maintained an excellent reversible capacity of 870 mAh·g^−1^ (0.53 mAh·cm^−2^) after 80 cycles, which is even much higher than the theoretical capacity of pristine Ni_3_S_2_ crystals. Such an amazingly capacity value can be ascribed to the nanoscale character of carbon wrapped Ni_3_S_2_ crystals and the integration of high conductive graphene, where the former provide more active sites for Li^+^ while the later enhance the electron transfer [37].

In addition to the high reversible capacity and cycle stability, the Ni_3_S_2_@C/RGO electrode exhibited also a superior rate performance (Figure 4d). The charge-discharge rates were evaluated at 100, 200, 500, 1000 and 5000, respectively and was then restored to 100 mA·g^−1^. Notably, with the increasing of the charge-discharge rates, the reversible capacities were measured to be about 920, 880, 850, 810 and 640 mAh·g^−1^, respectively. After the charge rate was back to 100 mAh·g^−1^, the electrode showed a discharge capacity of about 1150 mAh·g^−1^, which is even higher than the initial discharge capacity (920 mAh·g^−1^) The superior rate performance of Ni_3_S_2_@C/RGO composite can be attributed to the wrapping effect of the graphite carbon layer covering onto the Ni_3_S_2_ nanocrystals and the integration of the high conductive RGO substrates. The RGO sheets stacked into a porous structures can also act as a buffer matrices to alleviate the side effect originating from the volume changes of the Ni_3_S_2_ nanocrystals during the charging/discharging processes [19,38]. On the other hand, the wrapping effect of the carbon layer will protect further the Ni_3_S_2_ nanocrystals from the structural collapses due to the insertion/desertion of Li^+^.

The battery performance of Ni_3_S_2_@C/RGO electrode has been compared with those of Ni_3_S_2_ nanoparticles encapsulated in N-self-doped graphene sheets (Ni_3_S_2_@N-G) and other Ni_3_S_2_ electrode materials reported in previous literatures (see Table 1). It is obvious that the Ni_3_S_2_@C/RGO composite electrode shows a higher cycling performance at current density below 500 mA·g^−1^ and well rate capability at a current density of 5000 mA·g^−1^. A high capacity retention of 78% could be maintained when the current density soared from 200 to 5000 mA·g^−1^.

**Figure 5:**
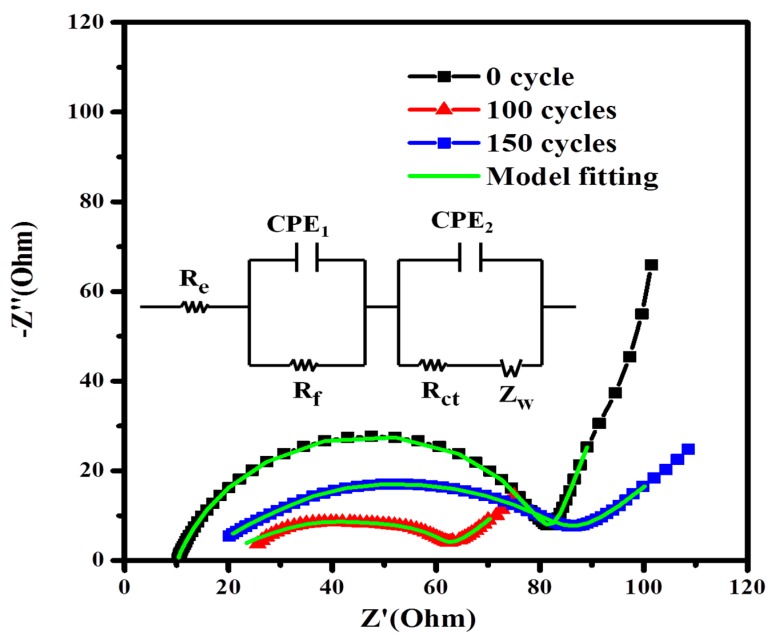
EIS curves of sample Ni_3_S_2_@C/RGO before and after different cycles and the fitting curves of the proposed equivalent circuit (inset shows the equivalent circuit).

Figure 5 shows the EIS curves of Ni_3_S_2_@C/RGO electrode before and after 100th and 150th cycles, along with the proposed equivalent circuit and fitting curve for samples. The intercept at the Z’ axis in the high-frequency region partially represents the resistance of electrolyte (*R*_e_). The high frequency semicircle can be assigned to the resistance (*R*_f_) and constant phase element (*C*_PE1_) of the SEI film; the medium frequency semicircle can be attributed to the charge transfer resistance (*R*_ct_) and constant phase element (*C*_PE2_) of the electrode/electrolyte interface [40,41]. The inclined line is related to the Warburg impedance (Z_w_) caused by Li ions diffusion in the electrode. In addition, the electrode reaction resistance decreased as the cycling processed, similar to that has been observed previously [42], due to the capacity increase on cycling. Furthermore, after the 100th to 150th cycles, the charge transfer resistance (*R*_ct_) increased upon cycling. It is probably due to the cracking and/or structural failure of the electrode caused by large volume changes [43].

Through the equivalent circuit and fitting curve, we obtained a series of analog resistance data listed in Table 2. As it shown that the Warburg impedance gradually decrease along with the cycles, because more ion channels were provided in the process of turning the electrode material from crystalline to amorphous.

### 3.5. Evolution of Capacity and Structure

It has been reported that Ni_3_S_2_-based anode materials deliver a decreasing reversible capacity against cycling due to huge volume changes, crystal growth and irreversible consumption of electrolyte materials [44]. Exceptionally, the Ni_3_S_2_@C/RGO sample of this study delivered an interesting reversible capacities which even increase gradually from 10 to 70 cycles (see Figure 4c).

The reversible capacity reached 665 mAh·g^−1^ at the 10th cycle and then increased to 870 mAh·g^−1^ at the 70th cycle. Similar behavior has been observed in graphene nanosheets [45], which was attributed to the lattice change of graphite during the Li^+^ insertion and extraction, generating more defects and active sites for Li^+^ storage.

**Figure 6:**
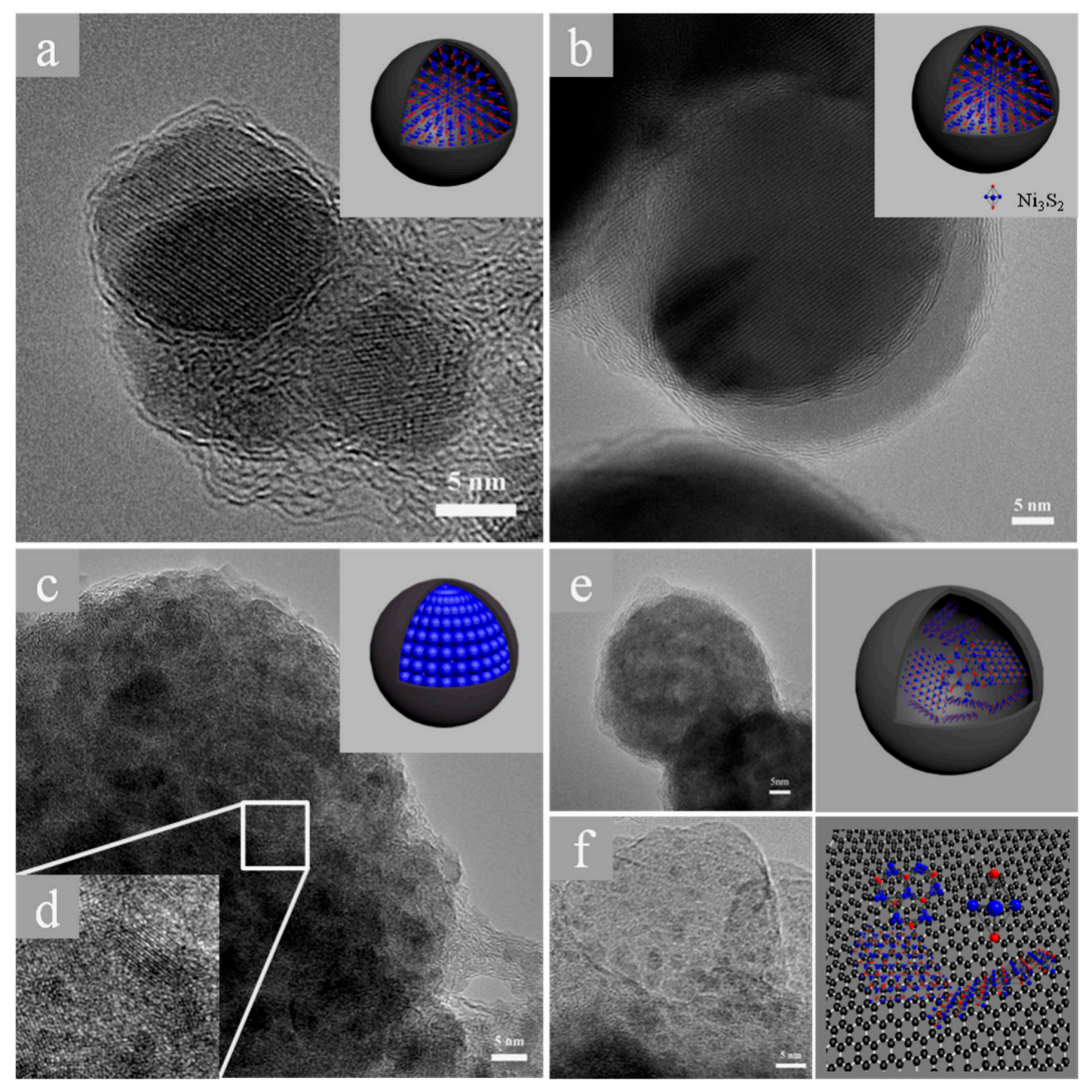
The diagrammatic of the nanocomposites during the Li^+^ insertion/extraction and their TEM images. (**a**) the TEM image of composite material without any cycling process; (**b**) the electrode material after 2 cyclings; (**c**) the TEM image after 20 cycles; (**d**) the HTEM image of image (**c**); (**e**,**f**) the different morphology after 60 cycles and some model image that insertion.

To investigate further the mechanism of capacity growth of the Ni_3_S_2_@C/RGO sample, ex-situ TEM and XRD methods were employed to characterize samples upon cycling. The ex-situ TEM image of Figure 6a shows a single Ni_3_S_2_ nanocrystal before cycling, which is wrapped tightly by a carbon layer. After the 2nd cycle, similar nanocrystal morphology is observed and single crystal wrapped with the carbon layer is maintained (Figure 6b). 20 cycles later, the crystallinity of the particle changed obviously and a transformation from a single crystalline phase to a polycrystalline phase can be clearly seen from Figure 6c and the enlarged image of Figure 6d. This change is attributed to the structural collapses of Ni_3_S_2_ single crystal with Li^+^ insertion/extraction. Nevertheless, the carbon layers could still wrap the polycrystalline Ni_3_S_2_ together as integration, meanwhile, the polycrystals provide additional active sites for redox reactions due to the explosion of more facets [46,47]. Such a structure change led to the increase of the reversible capacity and the decrease of the charge transfer resistance (*R*_ct_) upon cycles. After 60 cycles, two different morphologies are observed: that is, amorphous Ni_3_S_2_ particles are enclosed with a carbon layer (Figure 6e) and amorphous Ni_3_S_2_ particles without carbon layer but still anchored on the RGO sheets (Figure 6f). The release of the isolated amorphous Ni_3_S_2_ particles might be due to the cracking of the wrapping carbon layer. The amorphization of Ni_3_S_2_ crystals could create more active sites for lithium ions, corresponding to the fade-away of the charge plateau with an enhanced reversible capacity (Figure 4b). However, amorphous Ni_3_S_2_ without carbon coating led also an irreversible capacity.

Figure S6 shows the ex-situ XRD patterns of the composite electrode at different cycles. The gradual disappearance of the Ni_3_S_2_ characteristic peaks suggested that the crystalline structure of the Ni_3_S_2_ nanocrystals changed to amorphous one with cycling. Debye-Scherrer formula [48]

(1)  L=0.9λ/(B×cos (θ)) 

has been applied to illustrate the evolution of the size of Ni_3_S_2_ nanocrystals. Where *L* is the coherence length, *B* is the full-width at half-maximum of the peak, λ is the wavelength of the X-ray radiation and θ is the angle of diffraction. In the case of spherical crystallites, the relation between *L* and *D*, the diameter of the crystallite, is given by *L* = 3*D*/4. The particle size of the primary Ni_3_S_2_ particles derived from the Scherrer formula are of 35, 19 and 11 nm, respectively, for the Ni_3_S_2_@C/RGO composite cycled for 0, 5 and 20 rounds, respectively. The calculated data are quite close to the sized obtained by the ex-situ TEM observations (Figure 6).

## 4. Conclusions

In summary, Ni_3_S_2_ nanocrystals wrapped by carbon layers and inlaid on RGO sheets (Ni_3_S_2_@C/RGO) has been successfully fabricated by a simple spraying-assisted hydrothermal method. The carbon coated Ni_3_S_2_ nanocrystals delivered high specific capacity, excellent cycling stability and rate capacity. ex-situ TEM and XRD methods have been explored to elucidate the superior electrochemical performance of the composites by investigating the structure evolution of the Ni_3_S_2_ particles upon cycles. It has been found during the Li^+^ insertion/extraction processes, Ni_3_S_2_ nanocrystal were smashed to polycrystalline and turned gradually into amorphous phase but still enclosed by the carbon capsule. The wrapping effect of the carbon layer prevents the abruption of the Ni_3_S_2_ crystals upon Li^+^ insertion/extraction, exposing more active sites for electrochemical reaction with Li^+^ and improving the electrochemical performance of the composites.

## Figures and Tables

**Table 1:** Comparison of the electrochemical performance of Ni_3_S_2_@C/RGO with several reported Ni_3_S_2_-based materials.

Electrode Material	Published Year	Maximum Reversible Capacity (Current Density)	Cycle Number	References
Ni_3_S_2_@N-G	2014	809 mAh·g^−1^ (0.05 A·g^−1^) 490 mAh·g^−1^ (4.00 A·g^−1^)	150	[7]

Ni_3_S_2_ Nano flakes	2015	861 mAh·g^−1^ (0.40 A·g^−1^) 514 mAh·g^−1^ (4.00 A·g^−1^)	70	[1]

3D Ni_3_S_2_	2016	622 mAh·g^−1^ (0.15 A·g^−1^) 325 mAh·g^−1^ (1.20 A·g^−1^)	55	[39]

Ni_3_S_2_@C-RGO Slices	2016	520 mAh·g^−1^ (0.20 A·g^−1^) 410 mAh·g^−1^ (1.00 A·g^−1^)		[40]
Ni_3_S_2_@C/RGO	-	820 mAh·g^−1^ (0.20 A·g^−1^) 640 mAh·g^−1^ (5.00 A·g^−1^)	100	This work


**Table 2:** The evolution of the equivalent electrical circuit parameters along with the cycles.

Cycle Number	*R*_e_ (Ω)	*R*_f_ (Ω)	*R*_ct_ (Ω)	*Z*_w_(Ω)
0 cycle	10.2	74.0	39.1	88.5
100 cycles	18.1	40.2	34.3	47.2
150 cycles	15.2	75.2	78.4	34.2

## Supplementary Materials

The following are available online at http://www.mdpi.com/2079-4991/8/10/760/s1, Scheme S1: the reacting carbon coating processes of Ni_3_S_2_ assisted with CTAB, Figure S1: The XRD patterns of GO and RGO (a) and of sample Ni_3_S_2_@C/RGO thermally treated at different temperatures (b), Figure S2: The SEM images (a), (b) and TEM images (c), (d) of Ni_3_S_2_ without CTAB and with CTAB, respectively, Figure S3: SEM images of Ni_3_S_2_ (a), Ni_3_S_2_@C (b) and Ni_3_S_2_@RGO (c), Figure S4: TGA curve of Ni_3_S_2_@C/RGO, Figure S5: Raman spectra of Ni_3_S_2_@C-RGO and Ni_3_S_2_@C, Figure S6: The XRD pattern of electrode material after sever cycles, Figure S7: The SEM images of cellulose (a) and cellulose combine with GO (b), Figure S8: electrochemical impedance spectra of the Ni_3_S_2_@RGO and Ni_3_S_2_@C-RGO composites, Figure S9: The nitrogen adsorption-desorption isotherms and BJH pore size adsorption curves (the inset) of (a) Ni_3_S_2_@RGO composite and (b) Ni_3_S_2_@C-RGO composite.
