# Supplementary material for: Carbon Wrapped Ni3S2 Nanocrystals Anchored on Graphene Sheets as Anode Materials for Lithium-Ion Battery and the Study on Their Capacity Evolution
Source: Nanomaterials (Basel). 2018 Sep 26;8(10):760. doi: 10.3390/nano8100760 (PMC6215149; doi:10.3390/nano8100760)
Supplement: Supplementary file 1 [file nanomaterials-08-00760-s001.pdf]

nanomaterials

Supporting Information

Carbon Wrapped Ni_3S_2 Nanocrystals Anchored on Graphene Sheets as Anode Materials for Lithium-ion Battery and the Study on Their Capacity Evolution

Xianggang Guan[1,2], Xuehua Liu[1,2], Binghui Xu[1,2], Xiaowei Liu[1,2], Zhen Kong[1,2], Meiyun Song[2], Aiping Fu[2], Yanhui Li[3], Peizhi Guo[1,2], Hongliang Li[1,2,*]

[1] Institute of Materials for Energy and Environment, Qingdao University, Qingdao 266071, China

[2] College of Materials Science and Engineering, Qingdao University, Qingdao 266071, China

[3] College of Electromechanic Engineering, Qingdao University, Qingdao 266071, China

* Correspondence: lhl@qdu.edu.cn; Tel.: +86-159-5429-0135

The mechanism of carbon layer wrapping onto the Ni_3S_2nanocarstals

In the sample preparation process, Ni^{2+} solution was added into GO solution before mixing with Na_2S and CTAB. Because of the oxygen-containing groups on GO are negatively charged under a wide pH ranges, positively charged Ni^{2+}electrostatically interacted with the oxygen-containing functional groups on GO. Cellulose suspension can be prepared into nanosheets under spray-coagulation processing and combine well with GO sheets (**Figure S7**). Besides, cellulose will transform into amorphous carbon after calcination, which can not only improve the electrode conductivity (**Figure S8**), but work as a spacer between RGO layers to prevent RGO layers from restacking, as confirmed by N_2-sorption analysis (**Figure S9**). CTAB is an effective agent for dispersion of GO and is widely used for nanoparticles synthesis. [1] In this work, CTAB is employed as carbon source for wrapping Ni_3S_2 nanocrystals and an illustrative drawing to show the formation process of the composite is depicted in **Scheme S1.**The TEM measurements gave the evidence of carbon wrapping to the Ni_3S_2 nanocrystals (**FigureS2**). [2]

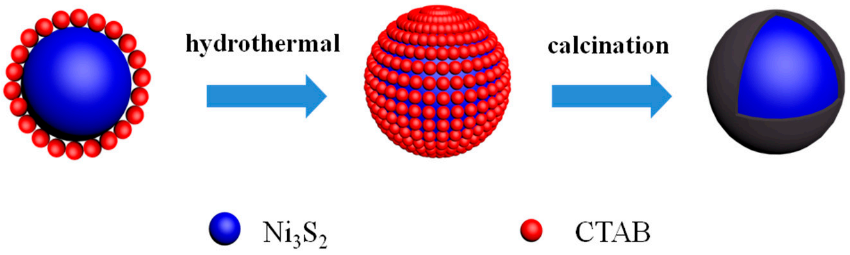

Scheme S1. The reacting carbon coating processes of Ni_3S_2 assisted with CTAB.

References

1. Li, Y.W.; Chen, C.C.; Wang, M.Y.; Li, W.Q.; Wang, Y.J.; Jiao, L.F.; Yuan, H.T. Excellent sodium storage performance of carbon-coated TiO_2 : Assisted with electrostatic interaction of surfactants. J. Power Sources.**2017**, 361, 326-333.

2. Liao, H.G.; Wu, H.; Wang, J.; Liu, J.; Jiang, Y.X.; Sun, S.G.; Lin,Y.H. Direct Electrochemistry and Electrocatalysis of Myoglobin Immobilized on Graphene-CTAB-Ionic Liquid Nanocomposite Film. Electroanalysis.**2010**, 22, 2297-2302.

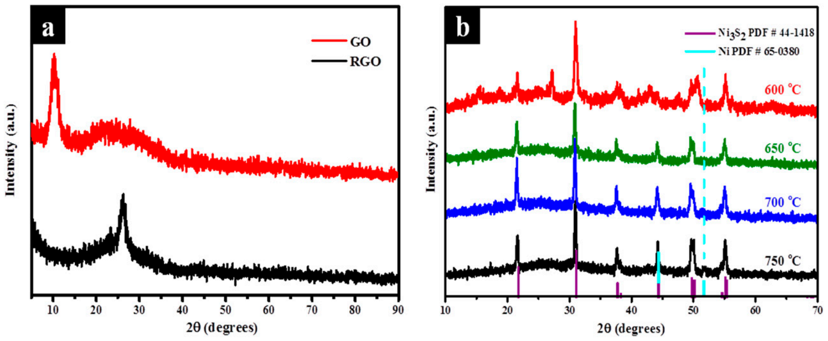

Figure S1. The XRD patterns of GO and RGO(a), and of sample Ni_3S_2@C/RGO thermally treated at different temperatures (b).

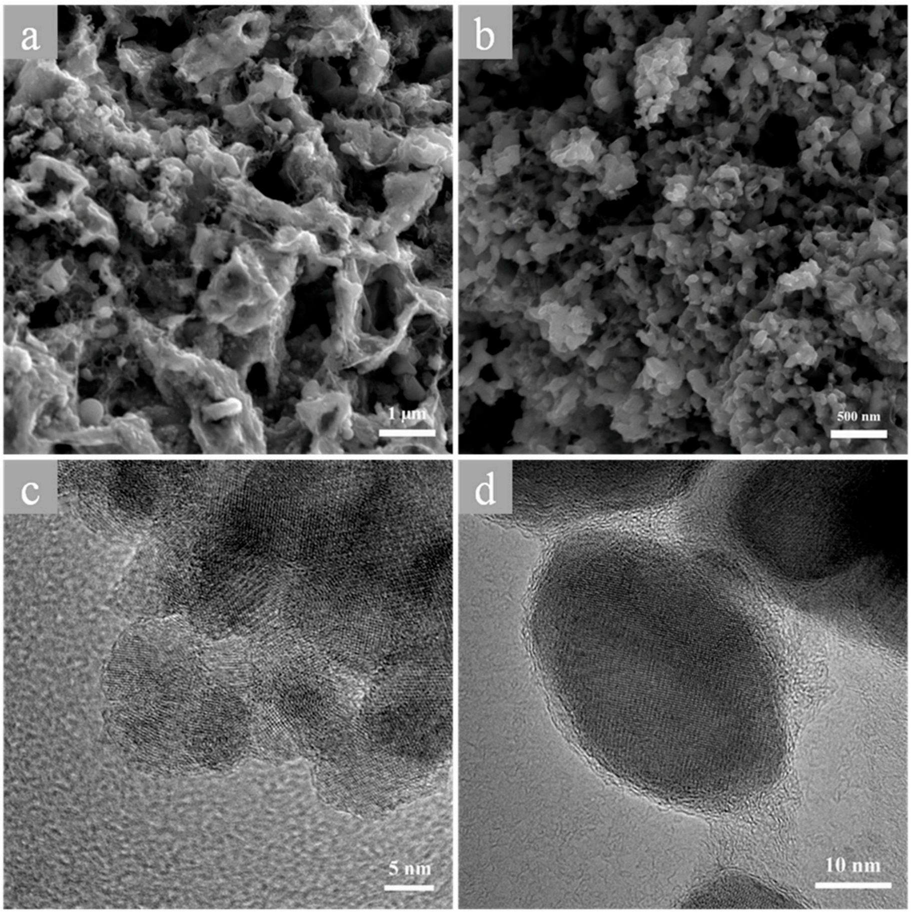

Figure S2. The SEM images (a), (b) and TEM images (c), (d) of Ni_3S_2 without CTAB and with CTAB, respectively.

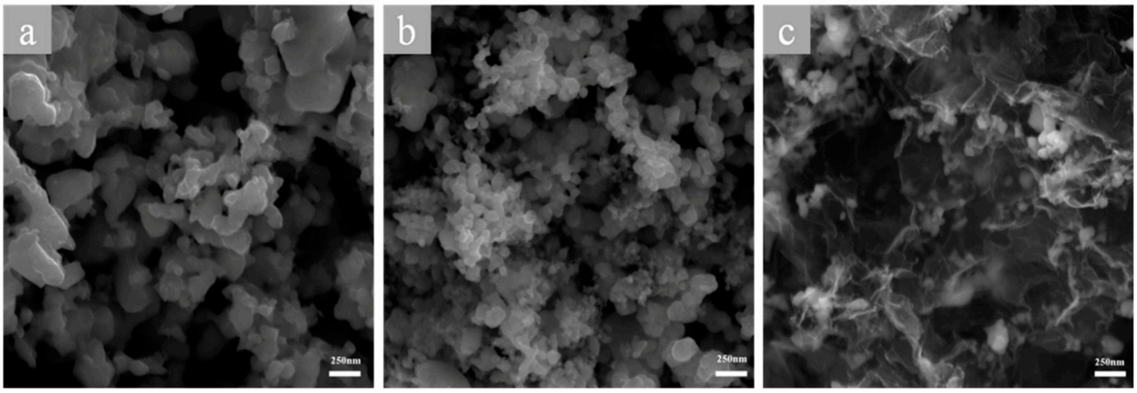

Figure S3. SEM images of Ni_3S_2 (a), Ni_3S_2@C (b) and Ni_3S_2@RGO (c).

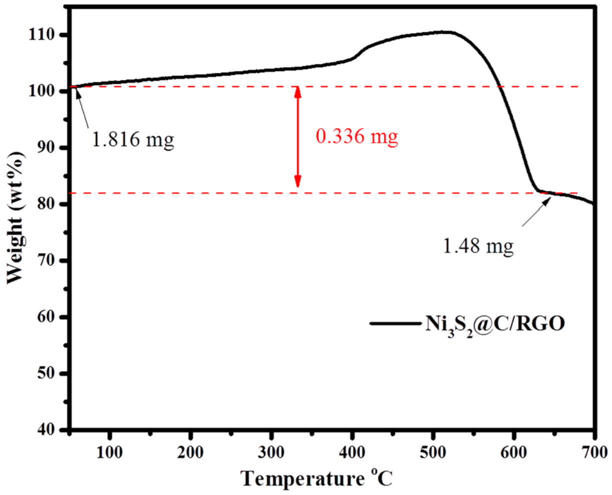

Figure S4. TGA curve of Ni_3S_2@C/RGO.

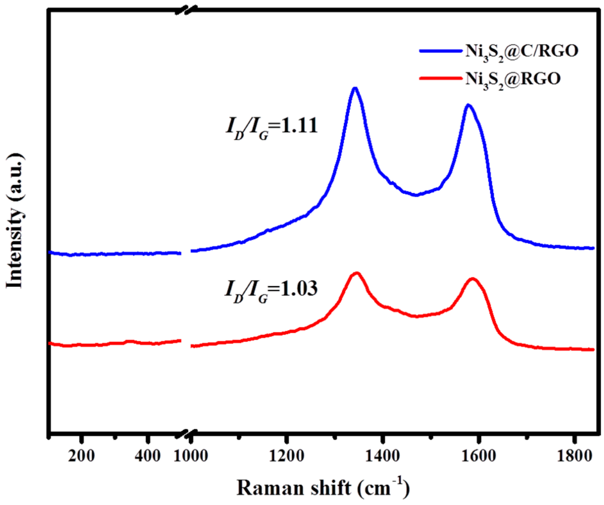

Figure S5. Raman spectra of Ni_3S_2@C/RGO and Ni_3S_2@C.

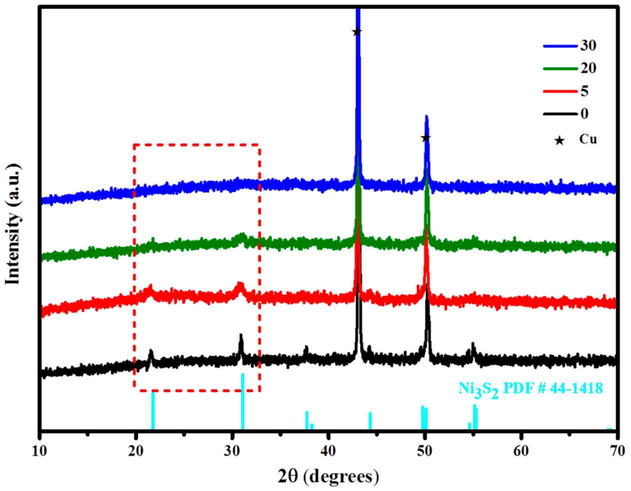

Figure S6. The XRD patterns of electrode material after sever cycles.

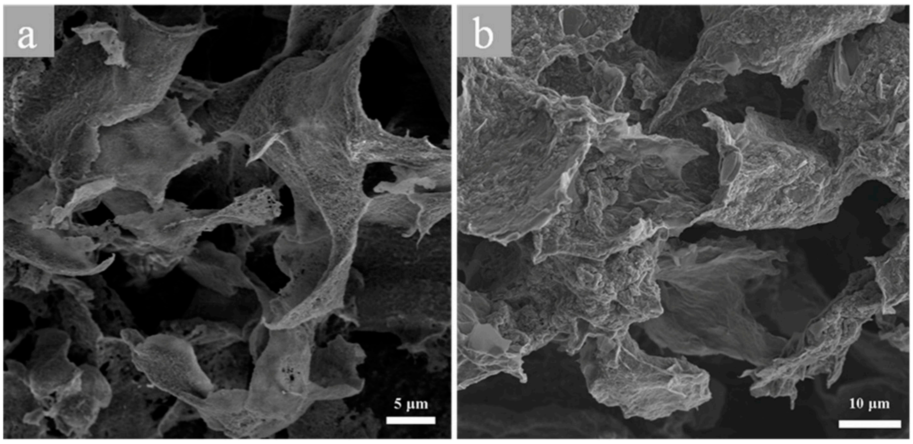

Figure S7. The SEM images of cellulose (a) and cellulose combine with GO (b).

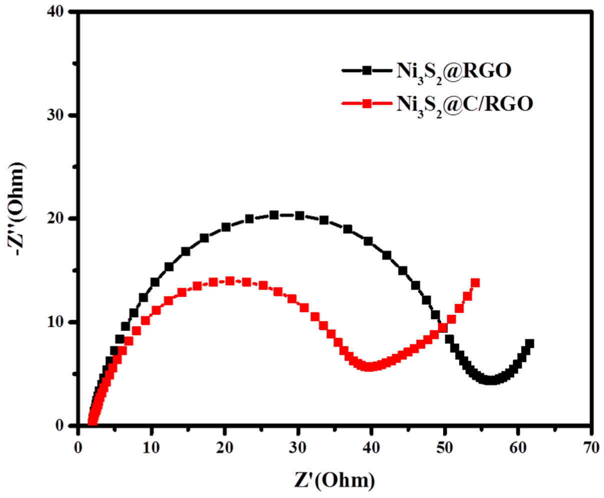

Figure S8. Electrochemical impedance spectra of the Ni_3S_2@RGO and Ni_3S_2@C/RGO composites.

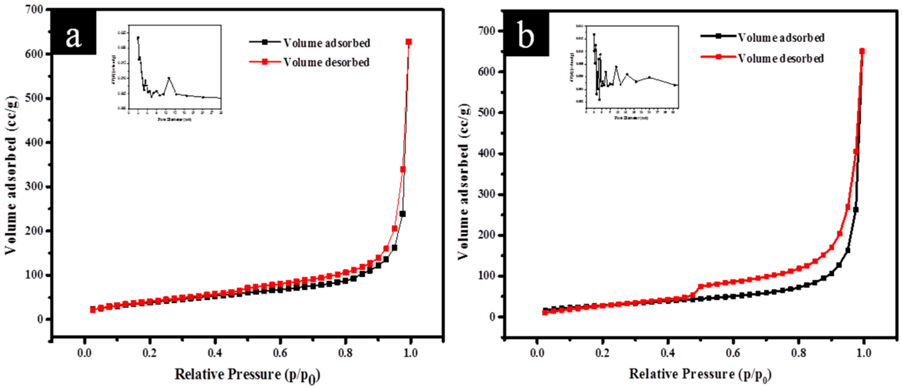

Figure S9. The nitrogen adsorption-desorption isotherms and BJH pore size adsorption curves (the inset) of (a) Ni_3S_2@RGO composite and (b) Ni_3S_2@C/RGO composite.
